# A Delayed Diagnosis of an Oral Foreign Body Due to Post-traumatic Neglect: A Case Report and Literature Review

**DOI:** 10.7759/cureus.95904

**Published:** 2025-11-01

**Authors:** Neetu Pansotra, Rupinder Kaur, Vishav Bandhu Jabalia

**Affiliations:** 1 Department of Dentistry, Oral and Maxillofacial Surgery, Government District Hospital, Pathankot, IND; 2 Department of Periodontology, Himachal Dental College, Sundernagar, IND; 3 Department of General Surgery, Government District Hospital, Pathankot, IND

**Keywords:** case report, child, foreign bodies, oral cavity, toothbrushing, wounds and injuries

## Abstract

Foreign bodies embedded in the oral cavity are infrequent and often pose substantial diagnostic challenges, particularly when the early traumatic incident occurs in childhood and remains unknown for years. This case report presents a 23-year-old female patient who reported chronic purulent discharge in the oral cavity. After a thorough investigation, the symptoms were linked back to an oropharyngeal injury that occurred when the patient was 11 years old after a fall while holding a toothbrush. When the orthopantomogram (OPG) failed to reveal any abnormalities, computed tomography (CT) was used for further evaluation, which revealed a retained toothbrush head lodged near the right pterygomandibular space. The foreign object was surgically removed with negligible postoperative complications, resulting in complete symptom resolution. When assessing persistent orofacial complaints of uncertain origin, this case emphasizes the value of a comprehensive patient history, early use of advanced imaging, and a high index of clinical suspicion. Additionally, it highlights the critical role of preventive education, particularly during routine dental visits.

## Introduction

Foreign bodies in the oral cavity may be presented through various mechanisms, including accidental trauma, ingestion, or iatrogenic causes. In children, these occurrences are not infrequent due to behavioral factors, limited awareness, and increased risk of falls or accidents. Foreign objects can remain embedded for prolonged periods, especially when initial trauma appears trivial, symptoms are mild, or access to specialized care is limited [1].

While metallic and radiopaque foreign bodies are frequently detected on routine radiographs, radiolucent materials such as plastic, wood, or rubber may dodge detection [2]. In such cases, the clinician’s dependence on patient history, meticulous clinical examination, and the judicious use of advanced imaging, such as computed tomography (CT), becomes critical. Cone-beam CT (CBCT) or magnetic resonance imaging (MRI) may further assist in delineating soft tissue involvement when radiolucent materials are suspected. Retained foreign bodies can present later with chronic inflammation, pain, trismus, discharging sinuses, or soft tissue swelling, often mimicking other odontogenic or non-odontogenic infections [2,3].

Despite being common in children, toothbrush-related injuries are frequently underreported because they are typically thought to be mild and self-limiting. When fragments of the toothbrush become embedded, particularly if radiolucent, the injury may remain undiagnosed for years. This under-recognition reflects both limited caregiver vigilance and the difficulty of detecting non-metallic objects on plain films. In the present case, the retained toothbrush head may have acted as a persistent nidus of infection, giving rise to chronic orofacial suppuration secondary to the childhood injury. The overlooked trauma and radiolucent nature of the foreign body likely contributed to the missed diagnosis, allowing low-grade infection and fibrosis to develop gradually over several years.

An uncommon occurrence of a plastic toothbrush head embedded in the oral cavity for 12 years after childhood trauma is presented in this case report. Even after multiple medical visits and ongoing symptoms, the foreign body was not discovered. Thus, this report illustrates that on evaluating chronic orofacial problems, general dentists should maintain a high index of suspicion, especially when dealing with patients who have a distant history of trauma. In order to prevent missed diagnoses, it also emphasizes the significance of comprehensive clinical evaluation, suitable imaging referrals, and interdisciplinary communication.

## Case presentation

**Figure 1:**
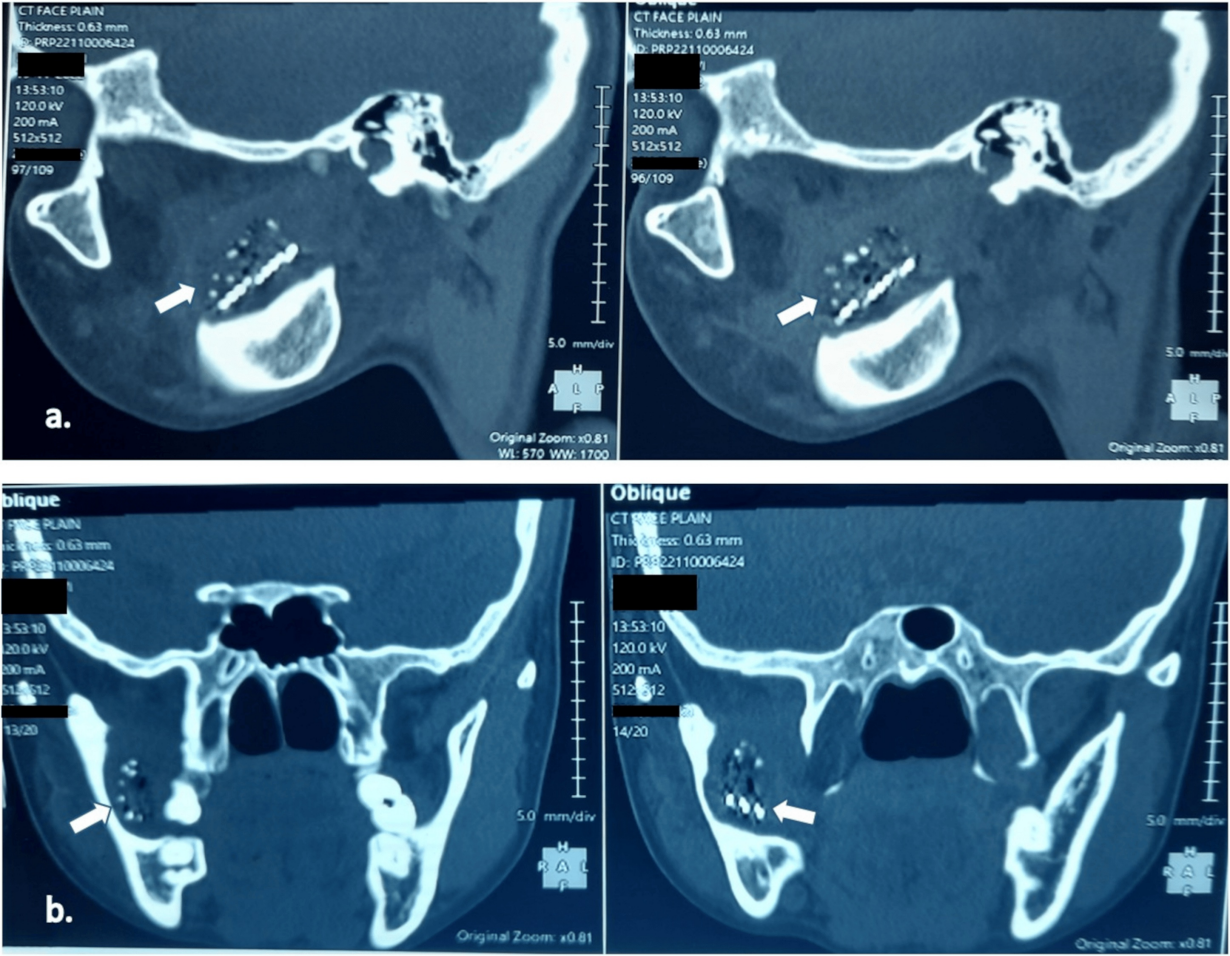
a: Oblique sagittal CT sections of the face demonstrating a hypodense foreign body with multiple linear projections (white arrows) in the right pterygomandibular region consistent with an embedded toothbrush head. b: Coronal CT sections showing the same hypodense structure (white arrows) extending from the oropharyngeal wall toward the right pterygomandibular space. CT: computed tomography

A 23-year-old female patient presented to the Dental Department of Government District Hospital with a one-month history of foul-smelling oral discharge and mild pus. Extraoral examination revealed swelling at the right mandibular angle. Intraoral examination showed frank pus posterior to the right mandibular first molar, with pain on palpation in the buccal vestibule. Inter-incisal mouth opening was restricted to <20 mm, suggesting trismus. Due to limited mouth opening, an orthopantomograph (OPG) was ordered, but showed no significant findings. A computed tomography (CT) scan was then performed, revealing a foreign body in the pterygopalatine space, with a small hypodense collection, surrounding inflamed soft tissue, and ill-defined bone resorption, consistent with chronic infection (Figure 1).

Upon detailed questioning, the patient recalled a fall at age 11 while brushing her teeth, causing significant oral bleeding. She was treated by a local physician who controlled the bleeding and prescribed antibiotics and analgesics for one week. Due to financial constraints, she did not seek dental care. Over the years, she experienced intermittent pain, swelling, and restricted mouth opening, which she tolerated. Despite multiple medical visits, the cause remained undiagnosed until recent pus discharge prompted her presentation. Based on CT findings, surgical extraction of the foreign body was planned. Preoperative antibiotics (amoxicillin 500 mg TID) and analgesics (ibuprofen 400 mg as needed) were prescribed for five days. Routine blood and urine tests were normal.

Timeline of events

The clinical sequence of events was as follows.

Injury

The patient had a fall while brushing at age 11, resulting in oropharyngeal trauma managed symptomatically by a local physician.

Symptom Onset

Intermittent swelling, pain, and restricted mouth opening developed gradually over several years.

Diagnostic Steps

Orthopantomogram revealed no abnormality; computed tomography detected a radiolucent foreign body within the right pterygomandibular space associated with chronic inflammation.

Intervention

The retained toothbrush head was surgically retrieved, followed by debridement under local anesthesia, gauze packing, and postoperative monitoring.

Outcome

There was uneventful healing and complete wound closure by four weeks, and inter-incisal opening improved from <20 mm preoperatively to approximately 38 mm postoperatively.

Surgical procedure

On the day of surgery, written informed consent was obtained from the patient. After aseptic preparation, local anesthesia (2% lidocaine with 1:100,000 epinephrine, 4 mL) was administered. An intraoral incision was made in the right mandibular buccal vestibule, extending from the distal aspect of the right mandibular first molar to the anterior border of the mandibular ramus. Blunt dissection, guided by preoperative CT, was done, and the pterygopalatine space was accessed through adjacent soft tissue.

**Figure 2:**
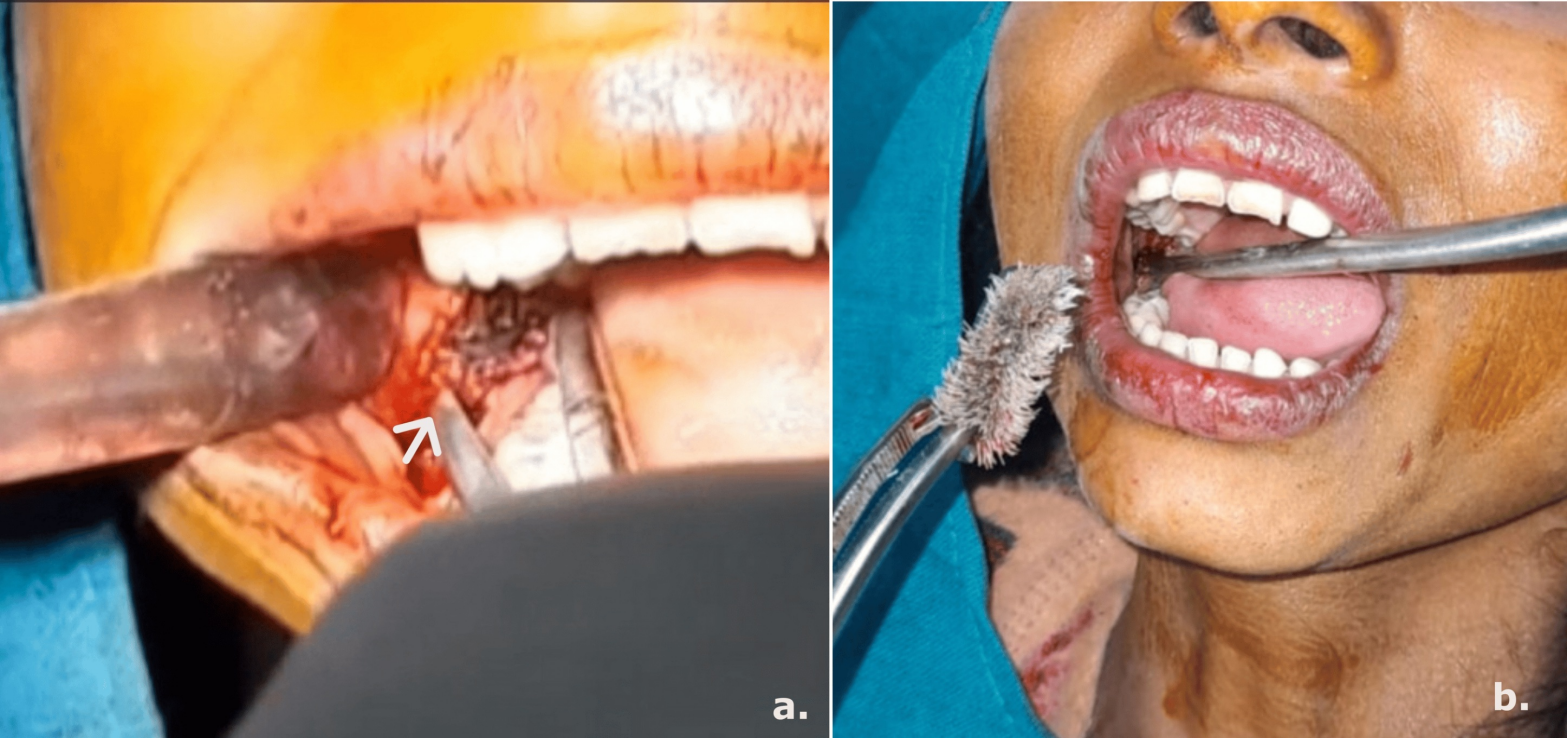
a: Intraoperative image showing retrieval of the toothbrush head with forceps (arrow). b: Image showing the retrieved toothbrush head following surgical removal.

The area was debrided with copious saline irrigation, and the foreign body was encountered, exposed, and retrieved (Figure 2a and Figure 2b).

**Figure 3:**
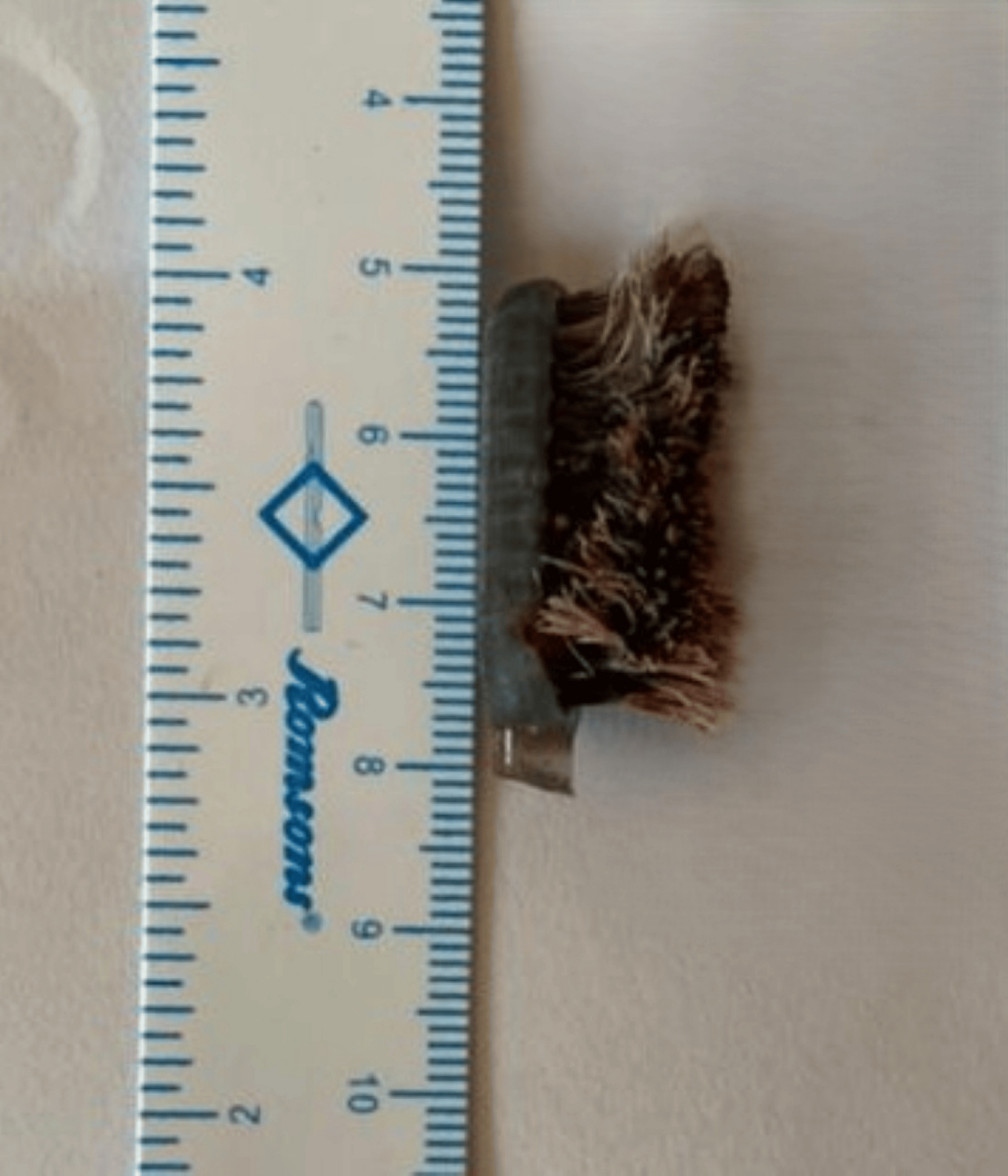
Image showing the dimensions of the retrieved toothbrush head (3.0×1.5 cm).

The object was identified as a toothbrush head measuring 3.0×1.5 cm (Figure 3).

**Figure 4:**
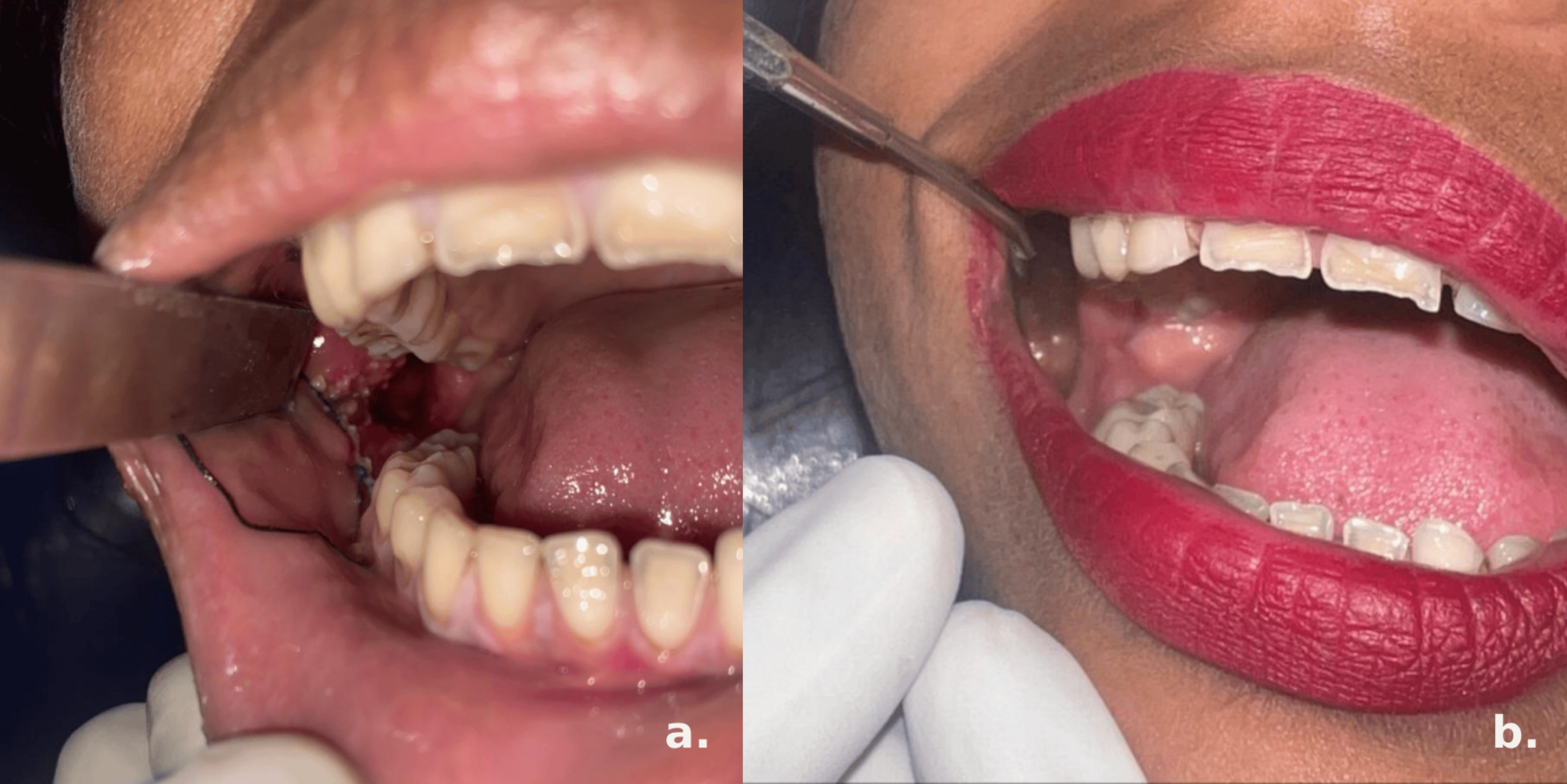
a: Postoperative intraoral image at 14 days showing an erythematous wound bed with reduced cavity size. b: Intraoral image 14 days after margin approximation, showing complete wound closure.

Due to the chronic wound’s size and depth, further debridement was performed, and the cavity was packed with iodoform gauze. The foreign body was retrieved intact with negligible blood loss, and no tissue specimen was sent for histopathological evaluation, as the object was clearly identified as a toothbrush head. The patient was admitted for seven days to monitor for infection, receiving ceftriaxone 1 g IV daily and paracetamol 1 g as needed. The dressing was changed on day 3 and monitored regularly. After 14 days, the patient came back with a raw and erythematous wound base containing granulation tissue, and the cavity dimensions had reduced, allowing for margin approximation (Figure 4a).

The margins were refreshed under local anesthetic infiltration and then approximated with resorbable sutures. The patient was recalled for follow-up after 14 days. At the 14-day postoperative visit, the wound showed complete closure with no complications, and healing was uneventful (Figure 4b).

## Discussion

Review of literature

A focused literature search was conducted using PubMed and Google Scholar databases up to May 2025. The PubMed search used combinations of MeSH terms and keywords using Boolean operators and filters to refine the results. 

The representative PubMed search string was as follows: ("foreign bodies"[MeSH Terms] OR "foreign body"[All Fields]) AND ("injuries and wounds"[MeSH Terms] OR "injury"[All Fields] OR "trauma"[All Fields]) AND ("oral cavity"[MeSH Terms] OR "mouth"[All Fields] OR "oropharynx"[All Fields]) AND ("toothbrush"[All Fields] OR "toothbrushes"[MeSH Terms]).

The Google Scholar databases were additionally searched with keywords that included “toothbrush,” “embedded,” and “oropharynx.”

For this review, we adopted the classification of toothbrush-related injuries as described in previous literature [4], categorizing them into blunt injuries, where there is no mucosal break or perforation; penetrating injuries, where there is deep laceration/perforation of the soft tissues, but the toothbrush has already been extricated from the wound at the time of presentation to the hospital; impalement injuries, where the toothbrush is impacted within the soft tissues because of “fish-hooking” of the bristles; and embedded injuries, when the head of the toothbrush breaks and is retained deep within the tissues.

The search was limited to articles reporting embedded or retained toothbrush head or parts within the oral or oropharyngeal tissues that required surgical removal. Articles and reports including indexed reviews, case series, and case reports published in English (or with English abstracts) in peer-reviewed journals and restricted to human studies were included.

Superficial injuries without retention, impalement injuries, or blunt trauma, or cases involving only external trauma were excluded to maintain specificity to embedded foreign bodies.

After screening titles and abstracts, 13 relevant full-text articles were identified and included for analysis (Table 1) [5-16].

**Table 1:** Summary of published cases (N=13) reporting embedded or retained toothbrush fragments in oral or oropharyngeal tissues requiring surgical removal. CT: computed tomography, OPG: orthopantomogram

Author(s)	Year	Age/sex	Site of injury	Duration	Imaging modality	Management approach	Complications
Tsukuda and Kudo [5]	2000	Infants	Back wall of meso-/hypopharynx	2 months	High-pressure contrast pharyngography (lateral/posterior anterior view)	Surgically removed	Stridor
Oza et al. [6]	2002	12 years/male	Pterygomandibular space	24 hours	X-ray	Surgically removed	Trismus
Tanaka et al. [7]	2002	12 months/male	Left posterior pharynx	Managed same day	Lateral and frontal X-ray of the neck/enhanced CT scan	Surgically removed	None
Burduk [8]	2006	18 months/male	Parapharyngeal space	Managed same day	Radiograph of the lateral neck	Surgically removed	Nil
Sasaki et al. [9]	2006	10 years/female	Upper oropharyngeal wall	Managed same day	CT	Operated under general anesthesia	Emphysema
Kumar et al. [4]	2008	35 years/male	Pterygomandibular space	11 months	OPG/CT scan	Surgically removed	Trismus and purulent discharge
Sathish et al. [10]	2011	5 years/female	Cheek	15 days	CT	Surgically removed	Extraoral sinus
Hennus and Speleman [11]	2011	2 years/male	Left lateral pharyngeal wall	Toothbrush head embedded for 1 day	CT angiography of the head and neck	Surgically removed	Internal maxillary artery pseudoaneurysm
Olajuyin and Okunola [12]	2012	8 years/male	Left tonsillar fossa	1 year	Plain radiograph	Surgically removed	None
Aregbesola and Ugboko [13]	2013	45 years/female	Left buccal space	10 weeks	Skull X-ray/oblique lateral skull X-ray	Surgically removed	Trismus
Goswami [14]	2016	4 years/female	Left parapharyngeal space	1 day	Axial CT scan of the neck	Surgically removed	Mild respiratory distress and fever
Kobayashi et al. [15]	2022	55 years/male	Electric toothbrush head in the left infratemporal fossa	4 days	CT scan	Surgically removed	Abscess formation in the masticatory space and trismus
Akisada et al. [16]	2024	73 years/male	Electric toothbrush head near the right submandibular gland	Managed same day	Cervical contrast-enhanced CT scan	Surgically removed	Laryngeal edema

Unintentional impalement or ingestion of foreign objects is commonly observed in young children, particularly following falls while holding items such as toothbrushes in the mouth [17]. Pediatric patients may not fully articulate their discomfort, which often results in delayed recognition. Of the 13 reported cases of embedded toothbrush fragments in the oral cavity identified in our review, the majority of cases occurred in pediatric patients, while only four occurred in adult patients. For more than 10 years, neither the patient nor the medical professionals noticed the long-term retention of the toothbrush head in the oral cavity, which was caused by a fall the patient suffered when she was 11 years old. The embedded object was discovered only after a comprehensive clinical and radiological evaluation. This highlights the necessity of greater clinical suspicion in dental and medical evaluations, especially in cases when symptoms are nebulous, persistent, or unresponsive to standard care.

Toothbrushes are among the most commonly implicated objects in oral cavity foreign body injuries in children, with numerous reports documenting impalement incidents involving the buccal vestibule [17]. However, cases of retained or embedded toothbrush fragments in oral tissues are rarely reported, although they may provoke chronic inflammatory reactions that can lead to complications such as granulomatous tissue formation or foreign body granulomas, fibrotic encapsulation, persistent fistulas, and trismus [18]. The toothbrush head in this instance was deeply buried in the buccal mucosa and was probably surrounded by fibrous tissue due to persistent, localized inflammation.

While more severe types, such as penetrating or impalement injuries, are typically identified earlier due to their acute sequelae, blunt oropharyngeal injuries brought on by toothbrush trauma are frequently overlooked because of their mild or absent clinical signs. Embedded injuries, in particular, pose a diagnostic challenge as the foreign object may not be identified during the initial clinical examination, only to later manifest as chronic complications [4,19]. In our reviewed cases, the duration of toothbrush retention within the oral cavity varied from as short as one day to as long as one year. By contrast, our case is notable for the exceptionally prolonged 12-year retention, representing a rare clinical scenario.

Despite the apparent simplicity of retrieving toothbrush-related injuries under local anesthesia, there are situations where such injuries might worsen and result in more complex complications. Trismus may result from localized inflammation, fibrosis, or mechanical interference with the masticatory muscles [6,13]. In the present case report, the presence of a foreign body in the pterygopalatine space could have led to restricted mouth opening or trismus by affecting the surrounding muscles. Our patient also reported trismus, which she noted had started to recover following surgical intervention with improvement in inter-incisal opening from <20 mm preoperatively to approximately 38 mm postoperatively. The pterygomandibular space, bounded laterally by the mandibular ramus and medially by the medial pterygoid muscle, embodies a probable fascial compartment where foreign bodies may remain undetected on routine radiographs due to superimposed bony structures.

There were notable challenges in establishing the diagnosis because non-metallic foreign bodies, such as plastic, are frequently radiolucent and may remain undetected on routine radiographs [20]. In this instance, computed tomography (CT) was pivotal in localizing the retained toothbrush head, whereas orthopantomography failed to reveal any abnormality. CT’s superior spatial resolution and three-dimensional capability make it the preferred modality for identifying radiolucent foreign bodies within complex maxillofacial spaces. Magnetic resonance imaging (MRI) can further aid in evaluating soft tissue changes or abscess formation when metallic interference is absent. Notably, two recent case reports describe embedded electric toothbrush heads following intraoral trauma: Kobayashi et al. [15] reported involvement of the infratemporal fossa, while Akisada et al. [16] documented a fragment extending from the oral floor to the submandibular space. In both instances, CT was essential for diagnosis and guided surgical management. Collectively, these cases underscore the diagnostic limitations of plain radiographs and highlight the indispensable role of cross-sectional imaging in evaluating chronic orofacial sepsis of uncertain etiology.

In this instance, the toothbrush head had entered the oropharyngeal area and stayed lodged as a foreign body, but neither the child nor the caregivers were aware of this. Only after a protracted 12-year period, when the purulent discharge persisted, was medical assistance sought.

## Conclusions

This case highlights the crucial importance of timely recognition and appropriate management of seemingly minor orofacial trauma to prevent delayed complications. Clinicians should maintain a vigilant approach when evaluating persistent or unexplained symptoms that may stem from previous injuries. A systematic diagnostic process, comprising comprehensive history-taking, detailed clinical assessment, and judicious use of advanced imaging, remains vital to prevent missed foreign bodies. The unusually prolonged diagnostic delay in this report underscores how overlooked childhood trauma can manifest as chronic orofacial infection years later. Preventive measures should include educating caregivers on safe oral hygiene practices, close supervision during toothbrushing, and discouraging movement while holding toothbrushes, thereby minimizing the risk of accidental oropharyngeal injury.
